# A decade-long trends in ligamentum flavum hypertrophy among spinal stenosis patients: A comparative analysis of incidence and patterns

**DOI:** 10.1016/j.jor.2025.05.056

**Published:** 2025-05-30

**Authors:** Waleed Albishi, Mohammed N. Alhuqbani, Omar A. Aldosar, Zyad A. Aldosari, Sara Alhomaidhi, Abdulrahman Alaseem, Waleed Awwad

**Affiliations:** Department of Orthopedic Surgery, College of Medicine, King Saud University, Riyadh, Saudi Arabia

**Keywords:** Ligamentum flavum, Lumbar vertebrae, Spinal stenosis

## Abstract

**Introduction:**

Lumbar spinal stenosis (LSS) is a prevalent degenerative condition, with ligamentum flavum hypertrophy (LFH) contributing significantly to neural compression. Despite its clinical importance, few studies have evaluated longitudinal trends in LFH. This study investigates the temporal progression and demographic associations of LFH over the past decade.

**Methods:**

This retrospective cohort study included adult patients undergoing surgery for LSS at a tertiary academic center during two periods: 2013–2017 and 2018–2023. Exclusion criteria included prior spinal surgery, trauma, malignancy, or inadequate imaging. Two radiologists reviewed axial MRI images to measure ligamentum flavum thickness (≥4 mm indicated hypertrophy). Demographic data and coexisting spinal conditions were also recorded.

**Results:**

A total of 202 patients were analyzed—98 from the initial and 104 from the later period. LFH prevalence rose from 24.5 % to 44.7 % over time. Patients aged ≥60 demonstrated significantly higher LFH rates in both periods (p = 0.004). Although female predominance persisted, it declined from 71.4 % to 55 %. LFH was most commonly associated with disc bulging, particularly at the same spinal level. Multilevel LFH increased from 25 % to 44.1 %, though L4–L5 remained the most affected segment.

**Conclusion:**

The incidence of LFH has increased substantially over the past decade, particularly among older adults. Given its role in LSS pathogenesis, greater attention to LFH in imaging and surgical planning is warranted. Further research is needed to clarify its etiology and guide effective management strategies.

## 1 Introduction

Affecting over 103 million people worldwide, lumbar spinal stenosis (LSS) is a common spine condition.^1^ Mostly affecting the elderly, this condition can cause incapacitating lower back and leg pain 2. This condition is defined by the narrowing of the spinal canal, resulting in compression of neuronal components, which may cause neurogenic claudication and radiculopathy. A primary contributor to this narrowing is ligamentum flavum hypertrophy (LFH), which plays an important role in both central and lateral recess constriction.^3^

The pathophysiology of LFH is not yet fully understood and is usually attributed to several risk factors. Together with mechanical stress and elastin degradation, fibrosis and chronic inflammation results ultimately in hypertrophy of the ligamentum flavum.^4^^,^^5^ The production of transforming growth factor (TGF)-beta by endothelial cells can induce fibrosis, mainly during the first phase of hypertrophy.^6^^,^^7^ Ultimately, these factors will result in the thickening of this band, which can be assessed, non-invasively, using magnetic resonance imaging.

Although LFH is clearly involved in the pathogenesis of LSS, longitudinal studies assessing the morphological development and temporal changes of this disorder over time have been scarce. Furthermore, changes in lifestyle, healthcare accessibility, the rising prevalence of obesity, and improved diagnostic capabilities over the past decade may have impacted the incidence, epidemiology, and distribution of LFH.

A decade-long investigation investigating the prevalence and distribution of ligamentum flavum hypertrophy will provide significant insights into the progression of this condition. This study seeks to uncover historical trends, examine potential demographic associations, and present updated data on the natural history of LF hypertrophy, thereby enhancing the understanding of spinal degenerative disease.

## 2 Materials and methods

### 2.1 Population

This retrospective comparative cohort study was performed in a tertiary academic institution. We targeted adult patients diagnosed with spinal stenosis who were scheduled for surgical intervention. We excluded patients with a history of spinal surgery before the inclusion period, those with a history of vertebral trauma, or malignancy, and patients with insufficient MRI image quality or incomplete medical records. The study comprised two temporal events separated by about a decade. The initial time set consisted of patients from 2005 to 2009, whereas the second time set included patients from 2016 to 2022, to allow the assessment of temporal patterns in LFH.

### 2.2 Data collection

At least two senior radiologists, blinded to patient identification and classification, assessed the MRI studies. Axial T1 and T2 weighted images were employed to assess the ligmentum flavum thickness bilaterally at each lumbar level from L1-L2 to L5-S1. The measurements were documented in millimeters. Patients who have LF thickness equal to or exceeding 4 mm were categorized into the LFH group. The hypertrophied segment's level was also reported. Additional spinal diseases, such as anterolisthesis, retrolisthesis, disc bulge, and degenerative changes, were also identified and reported. Demographic information of the selected sample were also obtained from patients' medical records.

### 2.3 Statistical analysis

Descriptive statistics were employed to summarize qualitative data, expressed in frequencies and percentages. All continuous variables were represented by the mean and standard deviation. Categorical data were analyzed using the chi-square test. A multivariate regression model was employed to ascertain determinants of ligamentum flavum hypertrophy, alongside other recognized spinal diseases. Temporal trends in LF thickness and distribution patterns across the decade were analyzed using time-stratified subgroup comparisons. A p-value <0.05 was considered statistically significant. Statistical analysis was performed using IBM SPSS Statistics, version 28.0 (IBM Corp., Armonk, NY, USA).

## 3 Results

The study included two hundred and two patients total—98 from the first period (2013–2017) and 104 from the second period (2018–2023). With a clear increase in incidence with time, ligamentum flavum hypertrophy (LFH) was found in 24 of these patients (24.5 %), in the first period and in 46 patients (44.7 %), in the later period (Table 1) (see Table 2).

**Table 1:** Demographic variables and incidence of LFH.

	First period (2005–2009)	Second period (2016–2022)
**Age (mean + - SD)**	50.5	53.35
**Gender – Females N (%)**	70 (71.4 %)	57 (55 %)
**LFH N (%)**	24 (24.5 %)	34 (44.7 %)

**Table 2:** Correlation between concomitant spinal ailments and incidence of LFH across two temporal periods.

	First period (2005–2009)		Second period (2016–2022)		P -value
	Normal LF	HLF	Normal LF	HLF	
**Anterolisthesis**	12 (50 %)	14 (18.9 %)	6 (17.6 %)	13 (30.9 %)	0.183
**Retrolisthesis**	2 (9.3 %)	3 (4.1 %)	1 (2.9 %)	2 (4.8 %)	0.685
**Disc bulge**	22 (64.7 %)	49 (66.2 %)	28 (82.4 %)	19 (45.2 %)	0.001
**Degenerative changes**	19 (79.2 %)	40 (54.1 %)	31 (91.2 %)	33 (78.6 %)	0.134

The average age of patients increased from 50.5 years in the initial period to 53.35 years in the second period. LFH was markedly more prevalent in patients aged 60 years and older. During the initial period, 36 % of individuals over 60 demonstrated LFH (20 out of 56), in contrast to 10 % (4 out of 42) among patients under 60 (p = 0.004). A similar age-based trend was noted in the subsequent period, with incidence rising to 61.3 % among individuals aged 60 and older. Female majority was noted in both periods, however the proportion declined from 71.4 % to 55 %.

In the initial cohort, disc bulging was observed in 71 cases, with 22 (31 %) of them exhibiting LFH. In 90.9 % of these cases, the LFH was at the same level as the disc bulge. Furthermore, 59 individuals demonstrated degenerative disc changes, with 19 (32 %) of them showing LFH; among them, 84.2 % were associated with the level.

Out of 23 patients diagnosed with spondylolisthesis, 20 had Grade 1. LFH was seen in all cases of Grade II and III as well as in 11 (55 %) of those suffering Grade I spondylolisthesis. One patient suffering from infected spondylitis also showed LFH. Only disc bulge showed a significant association with LFH (p = 0.001) during the second phase; the correlations with spondylolisthesis and degeneration were not statistically significant.

**Fig. 1:**
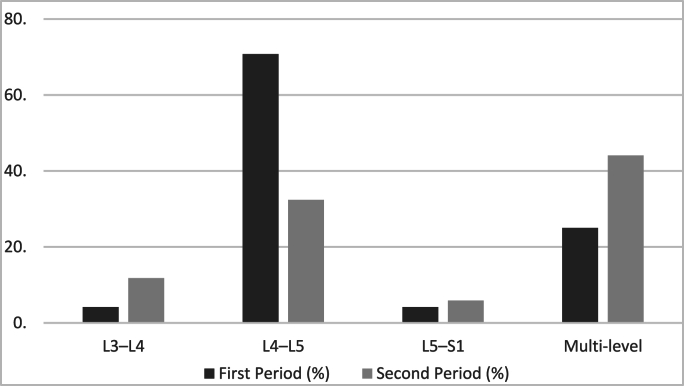
Comparison LFH between different spinal level across two temporal periods.

Of the LFH cases, 78 % were limited to one spinal level during the first period, mostly (70.8 %) at L4–L5 (Fig. 1). Twenty-five percent of patients showed multilevel involvement. Only minimal involvement at L3–L4 and L5–S1 was noted. On the other hand, during the second period multilevel LFH increased to 44.1 %, while L4–L5 remained the most often impacted individual level at 32.4 %.

## 4 Discussion

Our study showed a notable increase in the prevalence of ligamentum flavum hypertrophy (LFH), increasing from 24.5 % to 44.7 % during a ten-year period. This growth may indicate wider population-level trends, such as enhanced life expectancy, sedentary habits, and a growing global incidence of obesity.^8^, ^9^, ^10^, ^11^, ^12^ These causes induce continuous mechanical stress on the spine, acknowledged as a catalyst for ligamentous hypertrophy. Moreover, enhanced access to MRI and increased clinician awareness likely contributed to the more frequent and earlier identification of LFH. The results reflect same patterns noted in other degenerative spinal disorders, wherein diagnosis rates have risen due to technical progress and demographic changes.

Furthermore, our data indicate a distinct age-related rise in LFH prevalence, especially among persons aged over 60 years. This corresponds with prior research indicating that age-associated degeneration and elastin depletion in the ligamentum flavum result in heightened fibrotic thickening.^12^^,^^13^ Notably, although a female predominance was observed in both groups, it decreased from 71.4 % to 55 %, indicating a diminishing gender disparity in the manifestation of spinal degenerative diseases. This may be ascribed to occupational or lifestyle-related risk factors among males in the more recent cohort.

The correlation between LFH and disc bulge persisted as statistically significant throughout both time intervals, underscoring a probable biomechanical connection between intervertebral disc disease and ligamentous hypertrophy. A significant proportion of patients with LFH exhibited pathology corresponding to the disc bulge, supporting the idea that diminished disc height and annular degeneration may elevate stress on the posterior spinal structures, including the ligamentum flavum.^14^^,^^15^ While correlations with spondylolisthesis and overall degeneration were seen, they did not attain statistical significance in the subsequent cohort, potentially attributable to enhanced stratification or earlier imaging in the latter group.

A significant finding was the transition from primarily single-level involvement to a higher incidence of multilevel LFH in the more recent cohort. This may indicate more extensive degenerative changes resulting from cumulative mechanical stress or systemic risk factors, including diabetes and obesity.^9^^,^^16^ The L4–L5 segment is the most frequently affected level, aligning with its established biomechanical susceptibility as a transitional zone in the lumbar spine.^17^

Histologically, LFH is marked by elastin degradation, fibrosis, and an increase in collagen content. Transforming growth factor-beta (TGF-β), a cytokine involved in tissue remodeling, has been implicated in the early fibrotic phase of LFH.^13^^,^^17^^,^^18^ Chronic inflammation and mechanical microtrauma may further drive this fibrotic response. Our data, showing an increase in LFH incidence and distribution over time, lend support to these mechanistic theories and emphasize the need for further molecular research to validate potential therapeutic targets.

The rising incidence and multilevel distribution of LFH highlight the necessity for comprehensive preoperative imaging and planning from a surgical standpoint.^19^^,^^20^ LFH plays a significant role in the narrowing of the central canal and lateral recess, making its identification essential in decompression procedures. The possibility of under-recognition, particularly in early or unilateral cases, requires a heightened level of suspicion during evaluation. The results support tailored decompression strategies that consider the severity of LFH and its association with other degenerative conditions.^19^

This study has limitations. The retrospective design is susceptible to selection bias and may exhibit inconsistencies in MRI acquisition protocols over the past decade. Furthermore, although associations between LFH and degenerative findings were noted, causality cannot be determined. Future studies that include biochemical markers, histologic correlation, and long-term functional outcomes will provide significant insights. Prioritizing preventative strategies, including weight management, physical therapy, and early intervention for disc pathology, is essential.

## 5 Conclusion

In conclusion, this retrospective investigation indicates a notable increase in the incidence of LFH over the past decade. This underscores the necessity of performing additional research to comprehend the fundamental causes and effects of this pathology, considering its significant role in spinal stenosis. Surgeons must assess LFH to customize techniques that efficiently manage LFH and relieve strain on neural structures.

## CRediT authorship contribution statement

All authors contributed to the generation of research questions, study design, data analysis, manuscript writing, referencing, and final manuscript review.

## Patient/guardian consent

Not applicable. This study used retrospective, anonymized data and was exempt from informed consent requirements, as approved by the Institutional Review Board.

## Ethical statement

The study underwent review and approval by the Institutional Review Board.

## Declaration of interest

The authors report there are no competing interests to declare.

## Data availability statement

Data is publicly available upon request from the corresponding author.

## Funding statement

No funding was received for this study.

## Conflict of interest

The authors declare no conflict of interest.
